# King George's Fund for Sailors

**Published:** 1917-10-13

**Authors:** 


### KING GEORGE'S FUND FOR SAILORS.

In our issue of July 14 we drew attention to the establishment and organisation of this Fund, and we expressed for ourselves, and we are sure also for our readers, a full measure of goodwill towards the proposal. Our energetic and enterprising contemporary, the *Daily Telegraph*, has decided to institute a "special appeal" on behalf of the Fund, and we are asked to do what in us lies to aid the publicity of this enterprise. To such a request we gladly accede, and we do not doubt that the undertaking will be carried through with the thoroughness and success which have attended similar ambitions favoured by the same fortunate auspices.

The Fund, our readers may remember, is intended to effect for sailors' charities what King Edward VII.'s Hospital Fund has accomplished for our hospitals. It will make these charities better known to the public, and will claim for them sympathy and interest and help. By its influence it will encourage them to a higher level of efficiency and of successful and economical management. They will be set before the public eye as supervised and guaranteed by an impartial and competent authority, and in this way they may hope to command contributions from a wider area. At the same time, there is no intention to interfere with subscriptions going direct to the individual institutions. On the contrary, each organisation will retain its independence and will continue—and doubtless with success—to appeal to those who have some special personal or traditional interest in its welfare. But the central Fund will be in a position to supplement deficiencies, to suggest developments and improvements, and generally to establish the various organisations on a secure and prosperous basis. The first distribution from this Fund is to take place at Christmas, and hence there is need that donations shall be intimated without delay. On every ground it is to be hoped that our sailor-men and their kith and kin will receive at the earliest opportunity a convincing exhibition of the gratitude of the nation for services which no words can measure. The seamen who have stood between ourselves and the horrors of invasion, and have done this at the peril of their lives, are essentially men of deeds. Quite rightly, the standard they apply to themselves they will apply also to others. Some of them have been broken in our defence, and not a few have died that we may live. The time has come for a response, and here is the opportunity. The response must be a practical one and must insure that none of our injured sailors, and no family deprived of its support and protector by the casualties of our naval defence, shall want the fair comforts of life. The nation in some sense has the sea in its blood, and knows by a sure instinct that here lie safety and honour. Well, we must pay the price of Admiralty. In great ships of war, in merchantmen, in machines below the sea, in destroyers, in patrols, in trawlers, in mine-sweepers, in fishing fleets, and the rest, our seamen have paid the price in suffering, in endurance, and in blood. The landsman is now asked to take his share. That share, though small and monetary, stands as a debt of honour, and its discharge means help and comfort for those who have suffered in a great cause and on behalf of each one of us. *Bis dat qui cito dat.*

